# Brief Report: Associations Between Pain, Mobility, Quality of Life, and Mental Health in People of Black Ethnicities With HIV in London

**DOI:** 10.1097/QAI.0000000000003938

**Published:** 2026-11-12

**Authors:** Tamzin Hull, Lourdes Dominguez-Dominguez, Lucy Campbel, Julie Fox, Lisa Hamzah, Michael Evangeli, Shema Tariq, Whitney Scott, Frank A. Post

**Affiliations:** a King's College Hospital NHS Foundation Trust, London, UK;; b Berkshire Healthcare NHS Foundation Trust, Slough, UK;; c King's College London, London, UK;; d Guy's and St Thomas' Hospital NHS Foundation Trust, London, UK;; e St George's Healthcare NHS Foundation Trust, London, UK;; f Royal Holloway University of London, Egham, UK; and; g Institute for Global Health, University College London, London, UK.

**Keywords:** pain, mobility, mental health, HIV, inequity

## Abstract

**Background:**

Chronic pain, and its relationship with physical function and mental health, remains underexplored in people of Black ethnicities with HIV who experience intersecting inequities that may affect the experience and impact of pain. We investigated associations between severity of pain, physical function, and mental health in these populations.

**Setting:**

This study was conducted in 3 HIV clinics in South London, the United Kingdom

**Methods:**

We conducted a cross-sectional study in adults of Black ethnicities with HIV. Multivariable logistic regression analysis was used to examine associations between mild and moderate-severe pain, physical function, and mental health.

**Results:**

We enrolled 398 participants (median age 52 years, 55% female); 16% reported mild pain and 25% moderate-severe pain. In multivariable analysis, moderate-severe pain (vs. no pain) was associated with moderate-severely impaired mobility (adjusted odds ratio [aOR] 72.6 [25.7–250]), moderate-severely impaired ability to self-care (aOR 16.4 [4.1–111]), moderate-severely impaired ability to perform usual activities (aOR 14.9 [6.3–37.4]), and poor mental health (aOR 9.2 [5.1–17.1]) and mild pain with mild and moderate-severe impairment of mobility (aOR 6.5 [2.5–17.8] and 5.2 [1.5–18.8], respectively), mild impairment in the ability to perform usual activities (aOR 4.8 [1.9–11.9), and poor mental health (aOR 2.4 [1.2–4.8]).

**Conclusions:**

In this sample of people of Black ethnicities with HIV, two-fifths reported pain. Pain, regardless of severity, was associated with mobility impairment, difficulties performing activities of daily living, and poor mental health. We recommend evaluation of pain in HIV clinical services and establishing robust pain management pathways to maximize quality of life.

## Abstract 2

Supplemental Digital Content is Available in the Text.

## INTRODUCTION

Chronic pain is defined as pain persisting for more than 3 months in the absence of recognized tissue injury. It is often nonspecific in nature, multifactorial in etiology, potentially debilitating, strongly associated with detrimental impacts on quality of life and emotional well-being, and associated with substantial health care and societal cost.^1,2^ An estimated 13%–34% of the UK general population is living with chronic pain while prevalence estimates among people with HIV are generally higher, ranging from 25% to 85%.^1,3^ Such pain is often widespread, with frequent involvement of joints, head, legs, and back, and may have musculoskeletal, neuropathic, and/or inflammatory etiologies,^4^ and frequently overlooked as a contributor to quality of life (QoL) in HIV clinical practice.

In the pre-antiretroviral therapy (ART) era, pain in people with HIV was often linked to the direct neuropathic effects of uncontrolled HIV infection or tissue damage from malignancy or opportunistic infections.^4^ Early ART regimens containing stavudine or zalcitabine contributed to pain through their neurotoxic properties.^4^ Despite widespread use of modern antiretrovirals (ARVs) and high rates of viral suppression, pain remains a common and pervasive concern among people with HIV.^1,2^ As the prevalence of pain increases with age,^1,2^ pain is becoming an increasingly relevant clinical issue in aging cohorts of people with HIV.

There are well-documented inequities in the assessment and management of pain in ethnically and racially minoritized groups.^5^ In addition, minoritized groups may be reluctant to seek care for pain due to intersecting stigma and discrimination with respect to HIV, pain, ethnicity, and sex.^6^ However, chronic pain, and its relationship with physical function and mental health, remains underexplored in people of Black ethnicities living with HIV. We investigated associations between severity of pain and physical function and mental health in people living with HIV of Black ethnicities living in London to improve the understanding of the effects of pain on QoL in these populations.

## METHODS

This is an analysis of data from the cardiovascular disease, kidney disease, and diabetes in people of African ancestry with HIV (CKD-AFRICA) study. The CKD-AFRICA study was conducted between September 2020 and January 2022 among GEN-AFRICA (a multicenter study of clinical and genetic determinants of kidney disease in people of self-reported Black-African, Black-Caribbean, or other Black ethnicities living with HIV in the United Kingdom) participants to identify clinical and sociodemographic factors associated with comorbidity and multimorbidity.^7^ Participants were aged between 30 and 65 years and had participated in a single study visit after providing written informed consent; they were reimbursed £25 for time and travel. The study was approved by a National Health Service Research Ethics Committee (20/LO/0946) and the Health Research Authority (IRAS 278244).

Participants self-completed a paper questionnaire that captured data on the presence of aches or pain lasting ≥1 day in the past month; quality of life using the validated EQ-5D-5L, which measures quality of life (QoL) as an index on a scale from 0 to 100 and in multiple domains (pain and problems with mobility, self-care, performance of usual activities, and anxiety or depression) using a 5-point Likert scale^8^; mental health symptoms using the validated Hospital Anxiety and Depression Scale (HADS)^9^; demographic data; and data on comorbidities, social determinants of health (SDoH), social isolation, stigma, and discrimination^7^ (Supplemental Digital Content, Table 1, https://links.lww.com/QAI/C730). These data were supplemented with HIV parameters obtained from clinical records.

Pain (our exposure variable) was categorized as no pain (no aches/pains lasting ≥1 day in the past month and/or no pain/discomfort today), mild pain (aches/pains lasting ≥1 day in the past month and slight pain/discomfort today), or moderate-severe pain (aches/pains lasting ≥1 day in the past month and moderate/severe/extreme pain/discomfort at present) (Supplemental Digital Content, Table 2, https://links.lww.com/QAI/C730).

Outcomes of interest were mobility, ability to self-care, and ability to perform usual activities, a priori categorized as unimpaired, mild impairment (reporting slight problems), or moderate-severe impairment (reporting moderate/severe problems). The mental health outcome was defined as a HADS anxiety or depression score >10 (Supplemental Digital Content, Table 3, https://links.lww.com/QAI/C730).

Logistic regression models were used to describe associations between pain status and outcomes of interest, with separate models evaluating associations between mild and moderate-severe pain and mild or moderate-severe impairments of physical function and poor mental health. Multivariable models were adjusted a priori for age and sex at birth, with additional covariates considered for inclusion if *P* < 0.1 in univariable analysis, as multiple SDoH were associated with pain and mental health in the study participants models were not adjusted for SDoH.^7^ A sensitivity analysis of the mental health outcome was conducted in which participants taking medications used to treat anxiety or depression were excluded. All statistical analyses were performed using R (R Foundation, Vienna, Austria; version 4.2.1).

## RESULTS

We enrolled 398 participants (218 [55%] women; median age of 52.3 years [interquartile range 45.4, 57.5]), with similar characteristics to those of GEN-AFRICA participants who were not enrolled (Supplemental Digital Content, Table 4, https://links.lww.com/QAI/C730). Of these, 235 (59%) reported no pain, 62 (16%) reported mild pain, and 101 (25%) reported moderate-severe pain. Among participants with mild and moderate-severe pain, 56% and 80%, respectively, reported pain for more than 1 year, 32% and 16% had missed days off work in the past month due to pain, and 33% and 50% had seen their primary care physician in the past month for their pain. Participants reporting pain were more likely to be female, have a higher body mass index (BMI), and experience social and economic marginalization (financial, food and job insecurity, loneliness/isolation, and discrimination/unfair treatment). HIV parameters, including time since HIV diagnosis and starting ART, nadir and current CD4 cell count, and virological suppression (HIV RNA <200 copies/mL) did not differ by pain status. Lung disease was the only comorbidity found to be associated with pain status (Table 1).

**TABLE 1.** Study Participants

		Overall	No Pain	Mild Pain	Moderate-Severe Pain	*P*
Demographic/clinical characteristics	N	398	235	62	101	
Age	Median [IQR]	52.3 [45.4–57.5]	52.0 [45.1–57.0]	51.4 [45.1–56.1]	54.4 [48.3–59.5]	0.06
Female sex	N (%)	218 (54.8)	109 (46.4)	40 (64.5)	69 (68.3)	**<0.001**
Time since HIV diagnosis (yr)	Median [IQR]	14.0 [9.5–18.0]	14.0 [9.0–18.0]	15.0 [10.0–19.3]	13.0 [9.5–18.5]	0.33
Time since starting ART (yr)	Median [IQR]	10.0 [7.0–15.0]	11.0 [7.0–14.0]	12.0 [7.5–15.0]	9.0 [6.0–14.0]	0.30
HIV RNA >200 copies/mL	N (%)	23 (5.8)	14 (6.0)	1 (1.6)	8 (7.9)	0.24
Nadir CD4 cell count	Median [IQR]	161 [70–276]	169 [73–283]	149 [41–223]	163 [74–275]	0.26
Current CD4 cell count	Median [IQR]	548 [372–749]	556 [368–750]	493 [362–717]	563 [397–754]	0.80
Body mass index (kg/m^2^)	Median [IQR]	30.0 [26.5–34.2]	29.7 [26.4–32.9]	31.8 [27.1–37.3]	31.0 [26.8–35.4]	**0.045**
Hypertension	N (%)	212 (53.3)	122 (51.9)	36 (58.1)	54 (53.5)	0.69
Diabetes mellitus	N (%)	74 (18.6)	43 (18.3)	11 (17.7)	20 (19.8)	0.93
Chronic kidney disease	N (%)	111 (27.9)	69 (29.4)	14 (22.6)	28 (27.7)	0.57
Cardiovascular disease	N (%)	33 (8.3)	15 (6.4)	7 (11.3)	11 (10.9)	0.25
Lung disease	N (%)	64 (16.1)	26 (11.1)	11 (17.7)	27 (26.7)	**0.002**
Cancer	N (%)	16 (4.0)	7 (3.0)	4 (6.5)	5 (5.0)	0.12
Social determinants of health						
Financial insecurity	N (%)	207 (52.3)	105 (45.1)	34 (54.8)	68 (67.3)	**0.001**
Food insecurity	N (%)	83 (22.3)	36 (16.4)	13 (22.4)	34 (35.8)	**0.001**
Housing insecurity	N (%)	44 (11.1)	20 (8.6)	8 (12.9)	16 (15.8)	0.14
Migration status insecurity	N (%)	48 (12.3)	26 (11.3)	8 (13.6)	14 (13.9)	0.77
Job insecurity	N (%)	113 (28.4)	50 (21.3)	18 (29.0)	45 (44.6)	**<0.001**
Low educational status	N (%)	124 (32.0)	67 (29.5)	19 (32.2)	38 (37.6)	0.35
Loneliness and isolation	N (%)	129 (32.5)	59 (25.2)	21 (33.9)	49 (48.5)	**<0.001**
Discrimination/unfair treatment	N (%)	124 (33.3)	53 (24.1)	23 (39.0)	48 (51.6)	**<0.001**
Clinical outcomes of interest						
Quality-of-life index score	Median [IQR]	80 [65–95]	90 [80–100]	75 [56–90]	60 [50–75]	**<0.001**
Impaired mobility	N (%)					**<0.001**
No impairment		271 (70.6)	207 (93.2)	39 (63.9)	25 (24.8)	
Mild impairment		42 (10.9)	9 (4.1)	15 (24.6)	18 (17.8)	
Moderate-severe impairment		71 (18.5)	6 (2.7)	7 (11.5)	58 (57.4)	
Impaired ability to self-care	N (%)					**<0.001**
No impairment		341 (88.8)	218 (97.8)	58 (96.7)	65 (64.4)	
Mild impairment		20 (5.2)	3 (1.3)	1 (1.7)	16 (15.8)	
Moderate-severe impairment		23 (6.0)	2 (0.9)	1 (1.7)	20 (19.8)	
Impaired in usual activities	N (%)					**<0.001**
No impairment		274 (71.4)	199 (89.2)	42 (70.0)	33 (32.7)	
Mild impairment		49 (12.8)	13 (5.8)	14 (23.3)	22 (21.8)	
Moderate-severe impairment		61 (15.9)	11 (4.9)	4 (6.7)	46 (45.5)	
Poor mental health	N (%)	109 (28.2)	32 (14.0)	19 (31.1)	58 (59.2)	**<0.001**

P values <0.05 is highlighted in bold.

Mild pain was defined as aches/pains lasting ≥1 d in the past month and slight pain/discomfort at present, and moderate-severe pain as aches/pains lasting ≥1 d in the past month and moderate/severe/extreme pain/discomfort at present; mild impairment was defined as reporting slight problems, and moderate-severe impairment as reporting moderate/severe problems; poor mental health was defined as a HADS anxiety or depression score >10 (Supplemental Digital Content, Tables 2 and 3, https://links.lww.com/QAI/C730).

In univariable analysis, those reporting moderate-severe pain had increased odds of reporting impaired mobility, difficulties with self-care or performing usual activities, and poor mental health (all *P* < 0.001, Table 2). These associations persisted after adjusting for age, sex, and clinical factors (as described in Supplemental Digital Content, Table 5, https://links.lww.com/QAI/C730); as compared with no pain, moderate-severe pain was associated with increased odds of moderate-severely impaired mobility (adjusted odds ratio [aOR] 72.6 [25.7–250]), moderate-severely impaired ability to self-care (aOR 16.4 [4.1–111]), moderate-severely impaired ability to perform usual activities (aOR 14.9 [6.3–37.4]), and poor mental health (aOR 9.2 [5.1–17.1]). Notably, mild pain was also associated with increased odds of mild and moderate-severe impairment of mobility (aOR 6.5 [2.5–17.8] and 5.2 [1.5–18.8], respectively), mild impairment in the ability to perform usual activities (aOR 4.8 [1.9–11.9), and poor mental health (aOR 2.4 [1.2–4.8]). The association between mild pain and poor mental health was minimally affected by the exclusion of 83 participants receiving medications to treat anxiety or depression (Supplemental Digital Content, Table 6, https://links.lww.com/QAI/C730).

**TABLE 2.** Associations Between Mild and Moderate-Severe Pain and Impairment of Physical Function and Mental Health

	Univariable Analysis					Multivariable analysis*				
	No Pain	Mild Pain	*P*	Moderate-Severe Pain	*P*	No Pain	Mild Pain	*P*	Moderate-Severe Pain	*P*
Impaired mobility										
Mild impairment	Ref	8.9 (3.7–22.4)	<0.001	16.6 (6.9–42.5)	<0.001	Ref	6.5 (2.5–17.8)	<0.001	17.7 (6.4–52.5)	<0.001
Moderate-severe impairment	Ref	6.2 (2.0–20,2)	0.002	80.0 (33.6–225)	<0.001	Ref	5.2 (1.5–18.8)	0.009	72.6 (25.7–250)	<0.001
Impaired ability to self-care										
Mild impairment	Ref	1.3 (0.1–10.0)	0.85	17.9 (5.7–78.6)	<0.001	Ref	ND		12.4 (3.4–60.0)	<0.001
Moderate-severe impairment	Ref	1.9 (0.1–19.9)	0.61	33.5 (9.5–214)	<0.001	Ref	ND		16.4 (4.1–111)	<0.001
Impaired in usual activities										
Mild impairment	Ref	5.1 (2.2–11.8)	<0.001	10.2 (4.8–22.8)	<0.001	Ref	4.8 (1.9–11.9)	0.002	10.0 (4.2–24.6)	<0.001
Moderate-severe impairment	Ref	1.7 (0.5–5.3)	0.37	25.2 (12.3–56.0)	<0.001	Ref	ND		14.9 (6.3–37.4)	<0.001
Poor mental health	Ref	2.5 (1.2–5.0)	0.009	8.9 (5.1–15.9)	<0.001	Ref	2.4 (1.2–4.8)	0.016	9.2 (5.1–17.1)	<0.001

* Adjusted for age, sex, and clinical factors (listed in Supplemental Digital Content, Table 5, https://links.lww.com/QAI/C730) with *P* <0.1 in univariable analysis.

ND, not done (N < 5 in each of these strata).

Results obtained by logistic regression; data shown are odds ratios with 95% confidence intervals; mild pain was defined as aches/pains lasting ≥1 d in the past month and slight pain/discomfort at present, and moderate-severe pain as aches/pains lasting ≥1 d in the past month and moderate/severe/extreme pain/discomfort at present; mild impairment was defined as reporting slight problems, and moderate-severe impairment as reporting moderate/severe problems; poor mental health was defined as a HADS anxiety or depression score >10 (Supplemental Digital Content, Tables 2 and 3, https://links.lww.com/QAI/C730).

## DISCUSSION

In this cross-sectional study of people of Black ethnicities living with HIV, we report, consistent with studies in other populations, a high prevalence of pain. We did not find an association between pain and HIV clinical outcomes. Regardless of severity, pain (including mild pain) was associated with mobility impairment, difficulties with performing usual activities, and poor mental health. Our results highlight that even mild pain, which is commonly overlooked and undertreated in clinical practice, may have substantial detrimental effects on QoL in these populations.

A relationship between pain and functional impairment is well established, both in the general population and among people with HIV.^2,10,11^ A previous US study of 1903 individuals with HIV (23% women, half racially/ethnically minoritized, and 42% with uncontrolled HIV) reported associations between moderate-severe pain and impaired mobility, ability to perform usual activities and self-care, and mood disorders but did not examine associations with mild pain.^12^ Chronic pain has also been associated with slower gait, falls, impaired physical function, and depressive symptoms in older people (mean age 61 years) living with HIV.^10^ Diminished physical function as a result of chronic pain also affects QoL, social life, and employment.^2,11^ The relationship between chronic pain and poor mental health is well established and likely bidirectional: Depression-related and anxiety-related symptoms can exacerbate the intensity and duration of pain, while persistent pain can lead to worse mental health.^13^ Our analysis builds upon previous work by also exploring associations with mild as well as moderate-severe pain and finding that pain is associated with a variety of physical and psychological impacts irrespective of severity.

Women living with HIV, especially of Black ethnicities, are more likely to experience chronic pain compared with their male counterparts, consistent with the findings of our analysis.^2,4^ Racial and gender inequities in the assessment and management of pain are widely recognized.^5^ For example, individuals of Black ethnicities are less likely to be prescribed medication, such as opioids, or to undergo therapeutic interventions such as orthopaedic surgery as part of chronic pain management, when compared with non-Black counterparts.^14^ Similarly, despite carrying a greater burden of chronic pain in both frequency and severity, women are less likely than men to receive sufficient or effective pain management or access appropriate services.^15^ These intersecting disparities contribute to a high burden of pain but limited access to appropriate interventions for Black women.

Furthermore, racially minoritized women living with HIV may be reluctant to seek care due to fears of disclosing their HIV status and face the intersectional stigma and discrimination relating to HIV, sex, ethnicity, and chronic pain.^6^ Perceived and internalized HIV stigma are associated with increased severity of pain, pain-related disability, and mood disturbance.^16^ Our findings also revealed a linear relationship between pain severity and reported experiences of discrimination and unfair treatment in personal settings. While underreporting of pain precludes appropriate management and may ultimately lead to poorer mental health, reduced QoL, and constrained capacity to engage in HIV care, we found no evidence of lower rates of suppression of HIV replication in our participants.^6^

Chronic pain is a complex biopsychosocial phenomenon, requiring both pharmacological and nonpharmacological approaches that address pain as well as physical, social, and psychological functioning.^17^ Given this complexity, its management must be systematic and multimodal. Health care professionals should adopt regular pain assessment into routine review while also assessing for impact on quality of life and encourage patients to access appropriate care. A range of effective management strategies should be made available to individuals experiencing pain and tailored, where possible, to their specific needs. Cognitive behavioral therapy (CBT) has a strong evidence base for managing pain in the wider population, and there is emerging evidence of the acceptability and potential efficacy of these approaches for people living with HIV and pain.^17,18^ Tailored HIV-specific pain resources, increased engagement within social networks, and peer support-based interventions may offer benefit to this population as well as social prescribing, which encourages participation in an array of group activities and links people to statutory services and other agencies for practical and emotional support, is emerging as an effective method to improve health and well-being outcomes in people living with chronic pain.^19,20^

Strengths of this study are the use of validated questionnaires to assess pain, mobility and mental health, social isolation, stigma and discrimination, and a well-characterized cohort of people of Black ethnicities living with HIV in South London, one of the most ethnically diverse and socioeconomically deprived areas in the United Kingdom. We acknowledge limitations, including a lack of information on the causes, chronicity, characteristics, and management of pain. The cross-sectional design precludes insights into the episodic nature of pain and direction of associations. The complex mechanisms by which pain affects physical and mental health care-seeking and lived experiences are worthy of further qualitative investigation, which could also elucidate acceptability of potential interventions.

In summary, nearly two-fifths of people of Black ethnicities living with HIV in our sample reported pain; this was associated with socioeconomic marginalization, impaired physical function, and poor mental health. Routine evaluation of pain and robust management pathways are essential if we are to maximize health, well-being, and QoL in this population and ensure equitable outcomes.

## Supplementary Material

## References

[1] Slawek DE. People living with HIV and the emerging field of chronic pain-what is known about epidemiology, etiology, and management. Curr HIV/AIDS Rep. 2021;18:436–442. 10.1007/s11904-021-00563-0 PMC8551001 34046859

[2] Sabin CA Harding R Bagkeris E . Pain in people living with HIV and its association with healthcare resource use, well being and functional status. AIDS. 2018;32:2697–2706. 10.1097/QAD.0000000000002021 30289809

[3] Fayaz A Croft P Langford RM . Prevalence of chronic pain in the UK: a systematic review and meta-analysis of population studies. BMJ Open. 2016;6:e010364. 10.1136/bmjopen-2015-010364 PMC4932255 27324708

[4] Addis DR DeBerry JJ Aggarwal S. Chronic pain in HIV. Mol Pain. 2020;16:1744806920927276. 10.1177/1744806920927276 PMC7252379 32450765

[5] Morais CA Aroke EN Letzen JE . Confronting racism in pain research: a call to action. J Pain. 2022;23:878–892. 10.1016/j.jpain.2022.01.009 PMC9472374 35292201

[6] Hobson JM Gilstrap SR Owens MA . Intersectional HIV and chronic pain stigma: implications for mood, sleep, and pain severity. J Int Assoc Provid AIDS Care. 2022;21:23259582221077941. 10.1177/23259582221077941 PMC8883374 35200070

[7] Dominguez-Dominguez L Campbell L Barbini B . Associations between social determinants of health and comorbidity and multimorbidity in people of black ethnicities with HIV. AIDS. 2024;38:835–846. 10.1097/QAD.0000000000003848 PMC10994070 38265411

[8] EuroQol. 2025. Available at: http://euroqol.org/information-and-support/euroqol-instruments/eq-5d-5l/.

[9] Zigmond AS Snaith RP. The hospital anxiety and depression scale. Acta Psychiatr Scand. 1983;67:361–370. 10.1111/j.1600-0447.1983.tb09716.x 6880820

[10] Derry-Vick HM Johnston CD Brennan-Ing M . Pain is associated with depressive symptoms, inflammation, and poorer physical function in older adults with HIV. Psychosom Med. 2022;84:957–965. 10.1097/PSY.0000000000001119 PMC9553263 35980785

[11] Madden VJ Parker R Goodin BR. Chronic pain in people with HIV: a common comorbidity and threat to quality of life. Pain Manag. 2020;10:253–260. 10.2217/pmt-2020-0004 PMC7421257 32484065

[12] Merlin JS Westfall AO Chamot E . Pain is independently associated with impaired physical function in HIV-infected patients. Pain Med. 2013;14:1985–1993. 10.1111/pme.12255 PMC3886835 24119077

[13] Uebelacker LA Weisberg RB Herman DS . Chronic pain in HIV-Infected patients: relationship to depression, substance use, and mental health and pain treatment. Pain Med. 2015;16:1870–1881. 10.1111/pme.12799 PMC4486324 26119642

[14] Anderson KO Green CR Payne R. Racial and ethnic disparities in pain: causes and consequences of unequal care. J Pain. 2009;10:1187–1204. 10.1016/j.jpain.2009.10.002 19944378

[15] eClinicalMedicine. Gendered pain: a call for recognition and health equity. EClinicalMedicine. 2024;69:102558. 10.1016/j.eclinm.2024.102558 PMC10937548 38486682

[16] Crockett KB Esensoy TA Johnson MO . Internalized HIV stigma and pain among women with HIV in the United States: the mediating role of depressive symptoms. AIDS Behav. 2020;24:3482–3490. 10.1007/s10461-020-02919-w PMC7669722 32418165

[17] Merlin JS Young SR Johnson MO . Intervention Mapping to develop a Social Cognitive Theory-based intervention for chronic pain tailored to individuals with HIV. Contemp Clin Trials Commun. 2018;10:9–16. 10.1016/j.conctc.2018.02.004 PMC5898474 29696153

[18] Williams ACdC Fisher E Hearn L . Psychological therapies for the management of chronic pain (excluding headache) in adults. Cochrane Database Syst Rev. 2020;8:CD007407. 10.1002/14651858.CD007407.pub4 PMC7437545 32794606

[19] Pilkington G Johnson MI Thompson K. Social prescribing for adults with chronic pain in the U.K.: a rapid review. Br J Pain. 2025;19:176–186. 10.1177/20494637241312064 PMC11752153 39850272

[20] Merlin JS Westfall AO Johnson MO . Cost-effectiveness of a chronic pain intervention for people living with HIV (PLWH). J Med Econ. 2018;21:122–126. 10.1080/13696998.2017.1377719 PMC6028014 28880698

