# Supplementary material for: Brief Report: Associations Between Pain, Mobility, Quality of Life, and Mental Health in People of Black Ethnicities With HIV in London
Source: J Acquir Immune Defic Syndr. 2026 Nov 12;101(12):1373–7. doi: 10.1097/QAI.0000000000003938 (PMC13764237; doi:10.1097/QAI.0000000000003938)
Supplement: Supplementary file 1 [file qai-101-1373-s001.docx]

**Table S1	Definitions of comorbidities and Social Determinants of Health**

| **Variable** | **Type** | **Definition** |
| --- | --- | --- |
| ***Comorbidities*** | | |
| **Systemic hypertension** | Composite | Mean of the second and third readings of systolic BP ≥140 mmHg and/or mean of the second and third readings of diastolic BP ≥90 mmHg and/or being on anti-hypertensive medication |
| **Diabetes mellitus** | Composite | HbA1c ≥6.5% and/or being on hypoglycaemic medication |
| **Chronic Kidney Disease** | Composite | eGFR <60 mL/min/1.73m^2^ and/or albumin/creatinine ratio >3 mg/mmol |
| **Cardiovascular Disease** | Composite | Arterial disease, defined as diagnosis of cardiovascular or cerebrovascular accident according to medical history or the stroke questionnaire; or a diagnosis of congestive cardiac failure, or venous disease, defined as having been diagnosed with deep venous thrombosis or pulmonary embolism |
| **Lung Disease** | Composite | Having a diagnosis of asthma, chronic obstructive pulmonary disease, or interstitial lung disease; or having symptomatic lung disease, i.e.: cough, phlegm, shortness of breath and/or wheezing on several or most days |
| ***Social Determinants of Health*** | | |
| **Financial insecurity** | Composite | Being behind with some or most bills, or not having enough money to meet basic needs or having enough money to meet basic needs only some of the time, or having needed financial support in the last year |
| **Food insecurity** | Simple | Having needed food support in the last year |
| **Housing insecurity** | Simple | Living in temporary accommodation, living with friends or family (lacking own accommodation), or being homeless |
| **Migration status insecurity** | Composite | Having temporary or limited leave to remain, or having no legal status in the UK, or having needed immigration support in the last year |
| **Job insecurity** | Composite | Being unemployed, sick, or disabled, or having needed employment support in the last year |
| **Low educational status** | Simple | The equivalent of O levels (age 16) or less |
| **Social isolation** | Composite | Being alone or isolated, defined as feeling often: a lack of companionship, left out or isolated from others, or lonely; or having no one to turn to for emotional support; or having needed support with loneliness/isolation in the last year |
| **Stigma and discrimination** | Composite | Feeling little or not at all fairly treated by friends, in dating situations, in starting a family or in their social life in general, or with regards to housing, education or job opportunities, or with regards to respect to their privacy or personal security and safety, or feeling avoided because of the HIV status |

**Table S2	Exposure of interest**

| 1. **Aches/pains in the past month** | |  |
| --- | --- | --- |
|  | Aches/pains that lasted for one day or longer (Yes/No) | |
|  | |  |
| 2. **Severity of aches/pains today** | |  |
|  | I have extreme pain or discomfort | |
|  | I have severe pain or discomfort | |
|  | I have moderate pain or discomfort | |
|  | I have slight pain or discomfort | |
|  | I have no pain or discomfort | |

**Analysis**

**No pain**: no aches/pains >1 day in past month and/or no pain or discomfort today

**Mild pain**: aches/pains >1 day in past month and slight pain or discomfort today

**Moderate-severe** **pain**: aches/pains >1 day in past month and extreme/severe/moderate pain or discomfort today

**Table S3	Outcomes of interest**

**Outcome: mobility**

I have no problems walking about

I have slight problems walking about

I have moderate problems walking about

I have severe problems walking about

I am unable to walk about

**Outcome: self-care**

I have no problems washing/dressing myself

I have slight problems washing/dressing myself

I have moderate problems washing/dressing myself

I have severe problems washing/dressing myself

I am unable to wash/dress myself

**Outcome: usual activities**

I have no problems doing my usual activities

I have slight problems doing my usual activities

I have moderate problems doing my usual activities

I have severe problems doing my usual activities

I am unable to do my usual activities

**Outcome: mental health**

Normal Hospital Anxiety and Depression Scale

Borderline Hospital Anxiety and Depression Scale

Abnormal Hospital Anxiety and Depression Scale

**Table S3 (continued)**

**Analysis:**

**Mobility**

**Mild impairment**: I have slight problems walking about vs. I have no problems walking about

**Moderate-severe impairment**: I have moderate/severe problems walking about/I am unable to walk about vs. I have no problems walking about

**Self-care**:

**Mild impairment**: I have slight problems washing/dressing myself vs. I have no problems washing/dressing myself

**Moderate-severe impairment**: I have moderate/severe problems washing/dressing myself/I am unable to wash/dress myself vs. I have no problems washing/dressing myself

**Usual activities**:

**Mild impairment**: I have slight problems doing my usual activities vs. I have no problems doing my usual activities

**Moderate-severe impairment**: I have moderate/severe problems doing my usual activities/I am unable to do my usual activities vs. I have no problems doing my usual activities

**Poor mental health**: Abnormal (>10) HADS score for anxiety and/or depression

**Table S4	Characteristics of participants in the parent GEN-AFRICA study who took part in the CKD-AFRICA study (and were included in the present analyses), and those who did not participate in the CKD-AFRICA study**

|  |  |  | **CKD-AFRICA participants (N=398)** | **GEN-AFRICA participants who did not participate in CKD-AFRICA (N=1359)** | **p-value** |
| --- | --- | --- | --- | --- | --- |
| **Age** | Years | Mean (SD) | 48.6 (8.4) | 47.8 (10.9) | 0.19 |
| **Sex** | Female | N (%) | 207 (54) | 744 (55) | 0.54 |
| **Region of ancestry** | African | N (%) | 305 (77) | 1086 (80) | 0.47 |
|  | Caribbean | N (%) | 78 (20) | 240 (18) |  |
|  | Mixed/Other | N (%) | 7 (2) | 17 (1) |  |
| **HIV risk** | Heterosexual | N (%) | 350 (90) | 1127 (83) | 0.02 |
|  | Other/unknown | N (%) | 48 (12) | 232 (17) |  |
| **Time since HIV diagnosis** | Years | Median (IQR) | 18 (13, 23) | 19 (14, 23) | 0.06 |
| **Nadir CD4 cell count** | Cells/mm^3^ | Median (IQR) | 222 (89, 365) | 211 (79, 336) | 0.14 |
| **Current CD4 cell count** | Cells/mm^3^ | Median (IQR) | 574 (414, 759) | 556 (384, 728) | 0.09 |
| **HIV RNA** | <200 copies/mL | N (%) | 366 (94) | 1251 (92) | 0.29 |

**Table S5	Co-factors included in the multivariable logistic regression models of**

- **Mild impairment of mobility**
  - **Age, Sex, BMI, Hypertension, Cardiovascular disease, Lung disease, Cancer, Poor mental health**
- **Moderate-severe impairment of mobility**
  - **Age, Sex, HIV RNA >200 copies/mL, BMI, Cardiovascular disease, Lung disease, Poor mental health**
- **Mild impairment of ability to self-care**
  - **Age, Sex, Cardiovascular disease, Poor mental health**
- **Moderate-severe impairment of ability to self-care**
  - **Age, Sex, Poor mental health**
- **Mild impairment of usual activities**
  - **Age, Sex, BMI, Hypertension, Diabetes mellitus, Cardiovascular disease, Chronic kidney disease, Poor mental health**
- **Moderate-severe impairment of usual activities**
  - **Age, Sex, HIV RNA >200 copies/mL, BMI, Cardiovascular disease, Lung disease, Poor mental health**
- **Poor mental health**
  - **Age, Sex, BMI, Lung disease**

**Table S6	Associations between mild and moderate-severe pain and impairment mental health (sensitivity analysis excluding participants on medications commonly used to treat anxiety or depression)**

|  | **Univariable analysis** | | | | | |  | | **Multivariable analysis*** | | | | | | |
| --- | --- | --- | --- | --- | --- | --- | --- | --- | --- | --- | --- | --- | --- | --- | --- |
|  | **no pain** | **mild pain** | **p** | **moderate-severe pain** | **p** |  | | **no pain** | | **mild pain** | **p** | **moderate-severe pain** | **p** |  |  |
|  |  |  |  |  |  |  | |  | |  |  |  |  |  |  |
| **Poor mental health** | ref | 2.9 (1.4, 5.8) | 0.003 | 7.7 (4.3, 14.2) | <0.001 |  | | ref | | 2.7 (1.3, 5.5) | 0.008 | 7.9 (4.2, 15.1) | <0.001 |  |  |
|  |  |  |  |  |  |  | |  | |  |  |  |  |  |  |
|  |  |  |  |  |  |  | |  | |  |  |  |  |  |  |
|  |  |  |  |  |  |  | | * adjusted for age, sex and clinical factors with p <0.1 in univariable analysis | | | | | | |  |
|  |  |  |  |  |  |  | |  | | | | |  |  |  |
